# Sea Surface Height Measurements Based on Multi-Antenna GNSS Buoys

**DOI:** 10.3390/s24113451

**Published:** 2024-05-27

**Authors:** Xiaoming Xue, Jichao Yang, Qing Zhao, Shengli Wang, Ranshuo Zhao, Hulin Shao

**Affiliations:** 1 College of Geodesy and Geomatics, Shandong University of Science and Technology, Qingdao 266590, China; xuexiaoming@sdust.edu.cn; 2 College of Ocean Science and Engineering, Shandong University of Science and Technology, Qingdao 266590, China; 202383190029@sdust.edu.cn (Q.Z.); shlwang@sdust.edu.cn (S.W.); zrs_ioe@sdust.edu.cn (R.Z.); shaohulin@sdust.edu.cn (H.S.)

**Keywords:** GNSS buoy, sea surface height measurement, multi-antenna GNSS attitude determination, attitude correction

## Abstract

Sea level monitoring is an essential foundational project for studying global climate change and the rise in sea levels. Satellite radar altimeters, which can sometimes provide inaccurate sea surface height data near the coast, are affected by both the instrument itself and geophysical factors. Buoys equipped with GNSS receivers offer a relatively flexible deployment at sea, allowing for long-term, high-precision measurements of sea surface heights. When operating at sea, GNSS buoys undergo complex movements with multiple degrees of freedom. Attitude measurements are a crucial source of information for understanding the motion state of the buoy at sea, which is related to the buoy’s stability and reliability during its development. In this study, we designed and deployed a four-antenna GNSS buoy with both position and attitude measurement capabilities near Jimiya Wharf in Qingdao, China, to conduct offshore sea surface monitoring activities. The GNSS data were processed using the Precise Point Positioning (PPK) method to obtain a time series of sea surface heights, and the accuracy was evaluated using synchronous observation data from a small sea surface height radar. The difference between the GNSS buoy and the full-time radar was calculated, resulting in a root-mean-square error (RMSE) of 1.15 cm. Concurrently, the attitude of the GNSS buoy was calculated using multi-antenna technology, and the vertical elevation of the GNSS buoy antenna was corrected using the obtained attitude data. The RMSE between the corrected GNSS buoy data and the high ground radar was 1.12 cm, indicating that the four-antenna GNSS buoy can not only acquire high-precision coastal sea level data but also achieve synchronous measurement of the buoy’s attitude. Furthermore, the data accuracy was also improved after the sea level attitude correction.

## 1. Introduction

The study of sea level changes is one of the important issues of concern in earth science and climate research today. With the gradual warming of the global climate, many disasters such as saltwater intrusion and flooding triggered by sea level rise have become an urgent challenge for the international community, especially for coastal countries and regions [1]. Although sea surface monitoring in most of the sea area can be covered by satellite radar altimetry [2], satellite altimetry is not able to accurately give the height of the sea surface in near-shore areas due to factors such as geophysical factors and the response of the instrument hardware [3,4], resulting in a large monitoring gap area in near-shore areas. With the gradual development and maturity of GNSS technology, ocean buoys equipped with GNSS receivers have become an important observation platform in the field of marine scientific research [5,6]. They are designed to continuously collect marine environmental information such as changes in the sea surface altitude to support marine forecasting and marine disaster warning [7,8]. Some scholars have attempted to carry GNSS receivers on sea buoys for sea level measurements [9]. In addition, there are also scholars who have utilized GNSS technology to calibrate satellite altimeters [10,11] and to monitor rivers and lakes [12,13]. In the last few years, research targeting GNSS buoys has become increasingly sophisticated, and the effective error of sea level measurements using GNSS technology can be as low as 1–2 cm compared to tide gauge stations and radar gauges [14,15].

In the marine environment, GNSS buoys will undergo multi-dimensional movements due to the influence of natural forces such as wind, waves, currents, etc. The accurate acquisition of attitude data is not only a key factor in evaluating the operational safety and reliability of the buoy and the equipment carried by them, but it is also the basis of the real-time and accurate calibration of the data collected by the buoy [16,17]. At present, the attitude measurement methods in the marine environment mainly include two kinds: inertial navigation technology and GNSS positioning technology [18,19]. Inertial Navigation Systems (INSs) with integrated gyroscopes and accelerometers provide highly accurate, high sampling rate acceleration and angular velocity information in a short period of time [20,21]. Scholars have already conducted experiments on board GNSS buoys [22,23]. However, with the accumulation of time, the gyroscope and accelerometer errors gradually accumulated, resulting in a serious shift in the positioning results, and due to the cost and the size of the buoy’s limitations, this buoy is mostly used in low-cost Miniature Inertial Measurement Units (MIMUs), is more likely to be affected by the temperature, vibration, and other factors, and measurements of the accuracy of the obvious decline is needed to fuse the other sensors to ensure the accuracy of its measurements [24]. The other method is GNSS-based attitude measurements, where the GNSS receiver can receive satellite signals to solve the buoy’s position and velocity information in real time [25]. In the case of carrying three or more GNSS antennas at the same time, carrier phase positioning techniques can be utilized to achieve highly accurate attitude measurements, provided that a minimum of five available satellites are ensured and the full circumferential ambiguity is successfully fixed. This measurement technology has been widely used in land and marine scenarios and has provided numerous solutions [26]. In addition, the applicability of multi-antenna GNSS technology on buoy platforms has also been extensively explored, and these explorations have confirmed the practicality of this technology on buoy platforms [27,28]. In this study, we designed an ocean buoy capable of carrying four GNSS antennas, and studied the application potential of multi-antenna GNSS technology in ocean buoys.

## 2. Methods and Materials

### 2.1. Multi-Antenna GNSS Buoy Design

**Figure 1:**
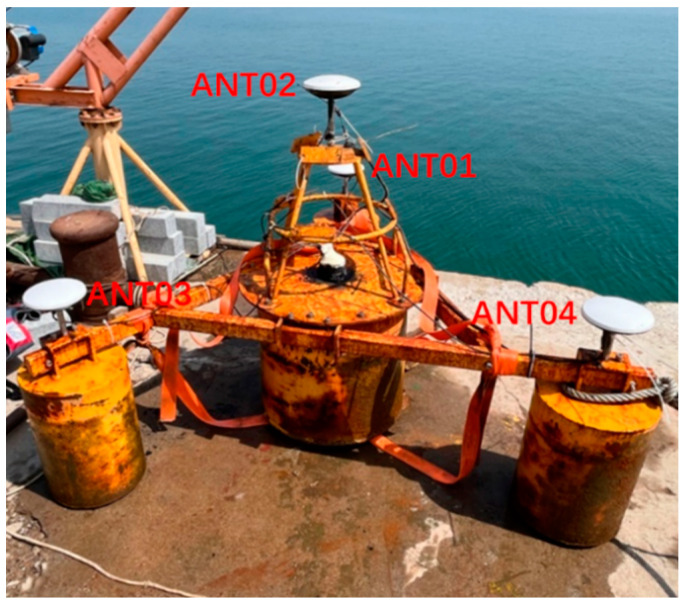
Multi-antenna GNSS buoy.

**Figure 2:**
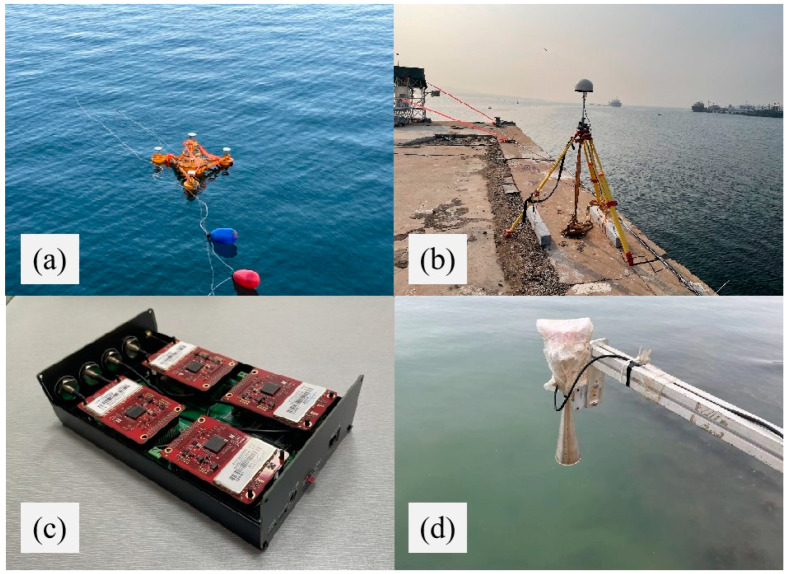
(**a**) Anchored GNSS buoy, (**b**) GNSS base station, (**c**) GNSS receiver embedded with UB482 navigation board, (**d**) small surface altimetry radar.

The multi-antenna GNSS buoy (Figure 1) as a whole consisted of a buoy top cover, a buoy body, three small floats, a support frame, and other structures. The main body of the buoy was an internal hollow metal drum, and the three floats were connected to the main body of the buoy by three connecting brackets to provide buoyancy support for the main body of the buoy. The design dimensions of the multi-antenna GNSS buoy are shown in Table 1. A GNSS antenna can be mounted above the buoy body and each of the three small floats. The GNSS receiver (Figure 2c) and power supply were installed in the empty compartment of the buoy body, and the GNSS cable was connected to the external GNSS antenna through the hole on the top of the buoy, and the hole and antenna connection was treated with special waterproof adhesive to improve the waterproof performance. A 4G antenna was mounted on a small platform on top of the buoy body, and the data collected by the GNSS buoy can be broadcast to the server via the network.

The GNSS receiver was embedded with four UB482 compact high-precision boards released by Unicore Communications, Inc. (Beijing, China). The boards are cost-effective, with low power consumption (2.4 W) and provide centimeter-level positioning accuracy, and can simultaneously collect observations in two frequency bands (GPS L1/L2, BDS B1I/B2I, GLONASS L1/L2, Galileo E1/E5b) at a maximum sampling rate of 10 Hz. The GNSS antenna adopted the HX-GPS500 antenna of Harxon Corporation (Beijing, China) which is small in size, has a strong shell, and the protection level reaches IP67. The GNSS antenna used in the experiment has certain out-of-band suppression capabilities, which can suppress unwanted electromagnetic signals. At the same time, the GNSS receiver also improves signal quality and suppresses interference through filters during the signal processing process. Furthermore, during the data processing phase, we pre-processed the observational values to remove satellites with poor quality and low elevation angles.

### 2.2. Experiment Introduction

**Figure 3:**
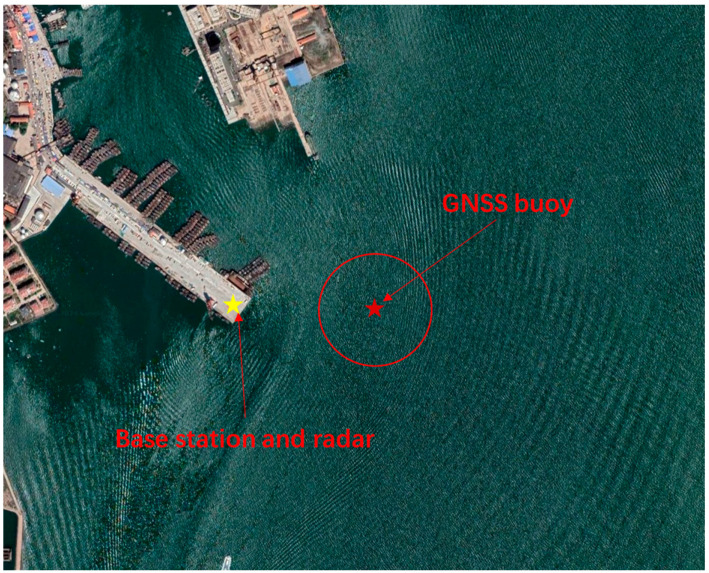
Location of Jimiya Wharf.

The experimental area is located near the Jimiya Wharf in Qingdao, China (Figure 3), and a small fishing harbor located on the west side of the pier, so we placed the buoys in the red area on the east side of the pier to try to avoid disturbances to the buoys caused by the fishing boats going out of the harbor. The GNSS buoys were deployed as their locations were close to each other, and a GNSS base station was set up in the yellow pentagram area of our pier (Figure 2b). In addition, since there is no tide measuring station near the dock, a small sea level altimeter was installed simultaneously in the yellow pentagon area (Figure 2d). The altimeter was fixed on the shore, and the antenna faced the sea surface for fixed-point observation. By measuring the distance between the altimeter and the sea level, the sea level height was calculated, which was used as a reference value to verify the accuracy of the GNSS buoy sea level measurement.

After deploying the total station, GNSS base station, and altimeter radar on the shore, we used the total station control point as a zero baseline, which was assumed to have an altitude of 0 m and a known fixed elevation difference from the Yellow Sea mean sea level baseline. First, we used the total station to accurately calibrate the elevation difference between the GNSS base station erection point and the altimeter radar deployment point relative to the zero datum. By adding these measured elevation differences to the assumed height of the zero reference point and applying the fixed elevation difference, the true elevation of these device points relative to the mean sea level of the Yellow Sea can be calculated. At the same time, after the buoys were in the water, we also took a ship to calibrate the actual draft depth of the buoys in the sea, and the results of the various calibrations are shown in Table 2.

### 2.3. GNSS Data Processing

**Figure 4:**
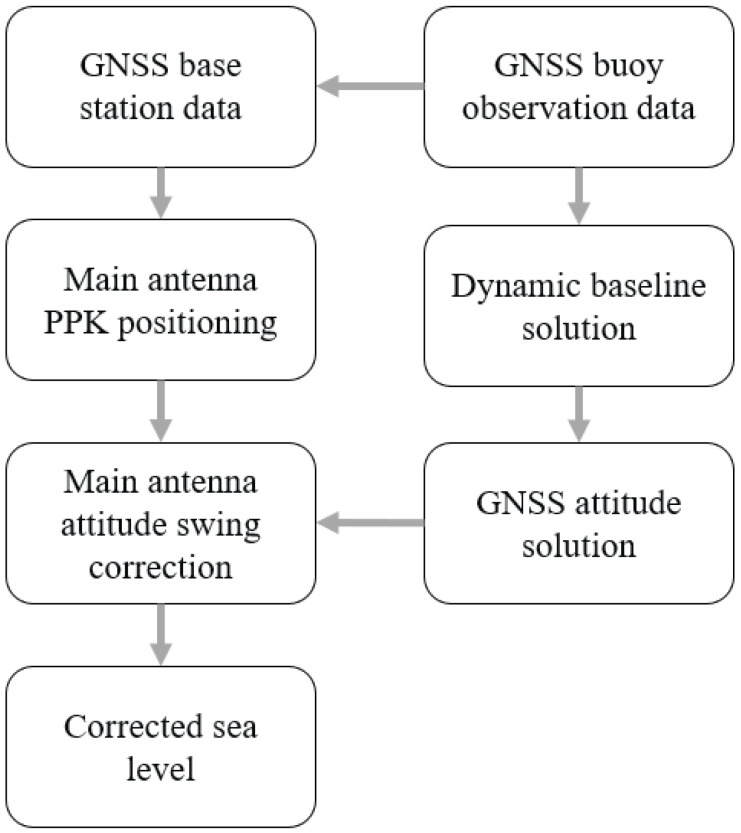
GNSS buoy data processing process.

The processing of the multi-antenna GNSS buoy data was divided into two parts. One part solves the high-precision positioning of the center antenna of the GNSS buoy to obtain the high-precision vertical displacement of the buoy, and the other part solves the attitude of the buoy by using the observation data of the multi-antenna GNSS. The process of data processing is shown in Figure 4. In this experiment, the GNSS buoy and GNSS reference station were deployed at the same time, and the distance between the two was about 30 m, which met the needs of high-precision PPK positioning. The observation data from the reference station and the GNSS buoy were used. The RTKLIB (ver.2.4.3) data processing software was used for the data processing of satellite observations in the L1 band of the three navigation systems, GPS, BDS, and Galileo, and the forward Kalman filter estimation was used in the data processing scheme. The vertical displacement of the buoy’s center antenna in the WGS-84 coordinate system was obtained by using the PPK positioning strategy [29].

**Figure 5:**
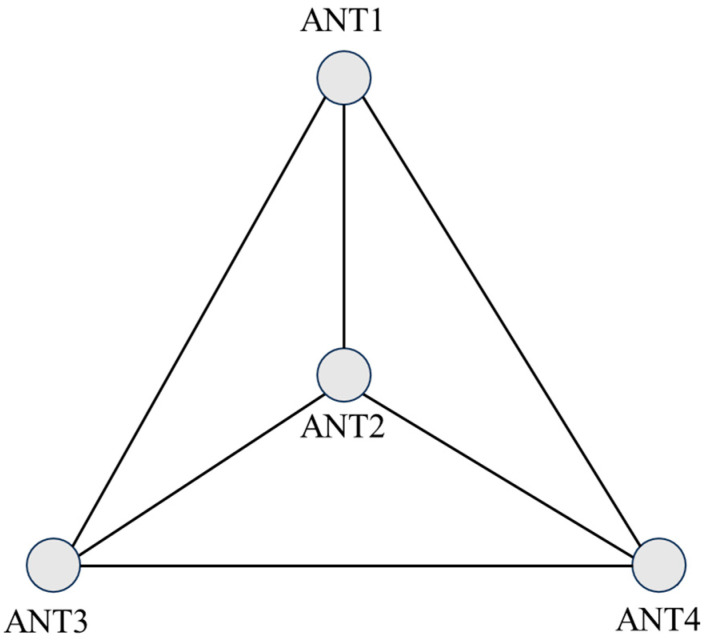
Multi-antenna GNSS buoy antenna configuration.

This experimental buoy carried four GNSS antennas, which are suitable for attitude solution using the least squares iteration method [30,31,32]. The antenna installation position on the buoy was fixed, in which the centrally located ANT02 antenna was the main antenna and the remaining three antennas were the auxiliary antennas, and the baseline vectors of the three auxiliary antennas relative to the main antenna under the carrier coordinate system were accurately measured using a total station before the experiment (Figure 5).

At the same time, the GNSS buoy can establish the main antenna as the center to build the carrier coordinate system, while the main antenna and the three auxiliary antennas constitute the baseline vector under the three carrier coordinate system. The solution of the baseline vector needs to be obtained by using the dynamic baseline solution; the dynamic baseline solution process is the same as the basic process of the PPK, with ANT02 as the base station, and ANT01, ANT03, and ANT04 as the mobile station. The dynamic baseline solution can be used to directly obtain the baseline vector information of the two antennas in the navigation coordinate system, and the relationship between the carrier coordinate system and the navigation coordinate system for any baseline vector can be expressed as follows:

(1) $$l_{i,n}=C_{b}^{n}l_{i,b}\left(i=2,3,L,m\right)$$

where $l_{n}$ and $l_{b}$ denote the baseline vectors of the baseline in the navigation coordinate system and carrier coordinate system, respectively, and $C_{b}^{n}$ denotes the attitude transfer matrix from the carrier coordinate system to the navigation coordinate system.

The baseline vectors in the carrier coordinate system are known at the time of installation, and the baseline vectors in the navigation coordinate system are obtained by solving for the baseline vectors, and solving for the attitude is solving for the three unknowns r, p, and y contained in the transformation matrix. Since least squares iteration requires the initial values of the given to be unknown, the attitude angles provided by the three-antenna attitude measurements can be used as the initial values $r_{0}$, $p_{0}$, and $y_{0}$. Bringing the initial values into Equation (1) allows the construction of the error equation for the attitude angle solution.

(2) $$v=l_{i,n}-C_{b}^{n}\left(r_{0},p_{0},y_{0}\right)l_{i,b}$$

By linearizing Equation (2), we can obtain Equation (3).

(3) $$v=l_{i,n}-C_{b}^{n}\left(r_{0},p_{0},y_{0}\right)l_{i,b}+\left[\begin{array}{ccc} \frac{\partial C_{b}^{n}}{\partial r}l_{i,b} & \frac{\partial C_{b}^{n}}{\partial p}l_{i,b} & \frac{\partial C_{b}^{n}}{\partial y}l_{i,b} \end{array}\right]\left[\begin{array}{c} \delta r\\ \delta p\\ \delta y \end{array}\right]$$

The content of Equation (3) is represented by different symbols, which can be obtained from

(4) $$A_{i}=\left[\begin{array}{ccc} \frac{\partial C_{b}^{n}}{\partial r}s_{li,b} & \frac{\partial C_{b}^{n}}{\partial p}s_{li,b} & \frac{\partial C_{b}^{n}}{\partial y}s_{li,b} \end{array}\right]$$

(5) $$\delta =\left[\begin{array}{c} \delta r\\ \delta p\\ \delta y \end{array}\right]$$

(6) $$B_{i}=C_{b}^{n}\left(r_{0},p_{0},y_{0}\right)$$

(7) $$s_{i}=l_{i,n}-C_{b}^{n}\left(r_{0},p_{0},y_{0}\right)l_{i,b}$$

Therefore, the attitude correction quantity δ can be expressed as

(8) $$\delta ={\left[\sum_{i=2}^{t}A_{i}^{T}{\left(Q_{s_{li,n}}+B_{i}Q_{s_{li,b}}B_{i}^{T}\right)}^{-1}A_{i}\right]}^{-1}\cdot \left[\sum_{i=2}^{t}A_{i}^{T}{\left(Q_{s_{li,n}}+B_{i}Q_{s_{li,b}}B_{i}^{T}\right)}^{-1}l_{i}\right]$$

The estimated value of the attitude angle is

(9) $$\left[\begin{array}{c} \hat{r}\\ \hat{p}\\ \hat{y} \end{array}\right]=\left[\begin{array}{c} r_{0}\\ p_{0}\\ y_{0} \end{array}\right]+\left[\begin{array}{c} \delta r\\ \delta p\\ \delta y \end{array}\right]$$

This process requires several iterations until the attitude correction δ is less than the threshold and the iteration is complete. 

According to the definition of the carrier attitude angle, the GNSS antenna wobble on the buoy is mainly caused by the attitude changes in the roll and pitch directions [33], and the correction of the GNSS buoy can be simply expressed as

(10) $$\Delta H_{t}=-x_{0}\times \sin\left(r_{t}\right)\cos\left(p_{t}\right)+y_{0}\times \sin\left(p_{t}\right)+z_{t}\times \left(\cos\left(r_{t}\right)\cos\left(p_{t}\right)-1\right)$$

where $\left(x_{0},y_{0},z_{0}\right)$ denotes the value of the pole arm of the GNSS antenna relative to the center of the buoy, and $r_{t}$ and $p_{t}$ denote the instantaneous roll and pitch.

## 3. Results

### 3.1. GNSS Buoy Trajectory

**Figure 6:**
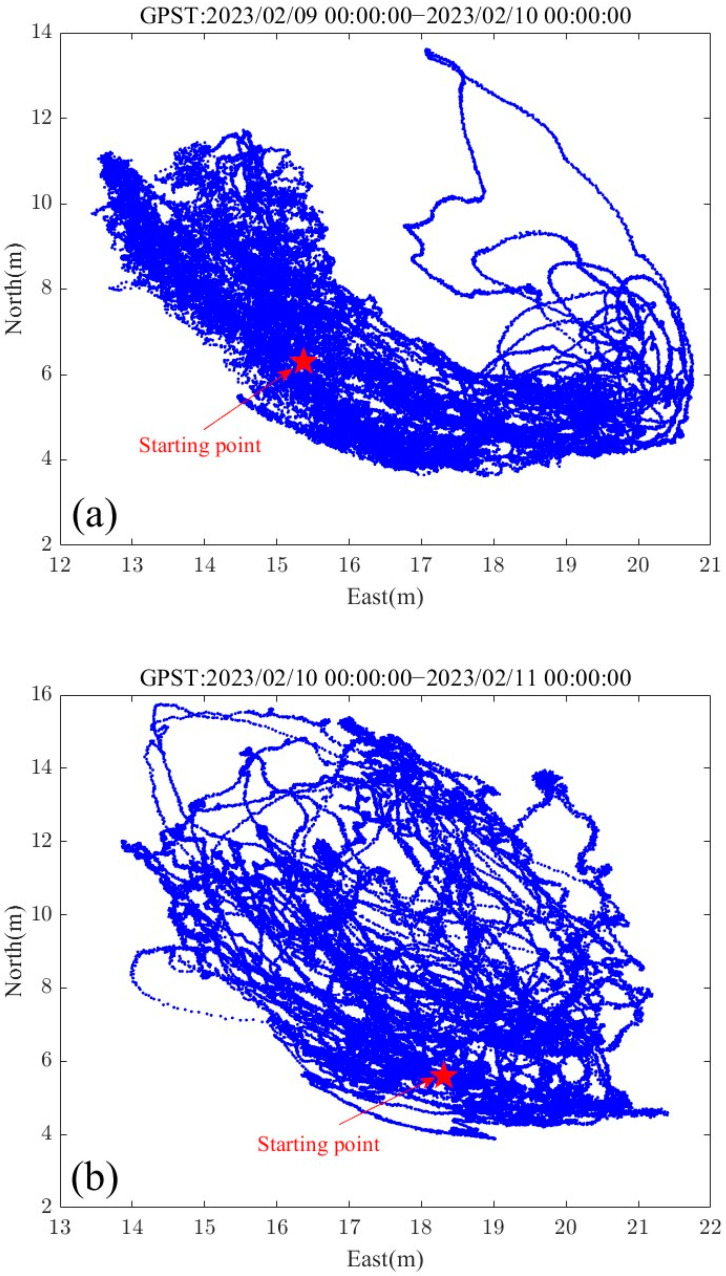
GNSS buoy planar motion trajectories: (**a**) 9 February 2023, 0:00–10 February 2023, 0:00; (**b**) 10 February 2023, 0:00–11 February 2023, 0:00.

The GNSS buoy was deployed in the anchored area for two days (9 February 2023, 0:00 to 11 February 2023, 0:00), and the data were processed using the PPK mode to obtain the planimetric trajectory of the buoy during these two days (Figure 6a,b). The maximum movement of the GNSS buoy in the east–west and north–south directions was about 10 m under the constraints of the anchor chain. On both days, the GNSS buoy rotated around the anchor point, and from 0:00 to 20:00 on 9 February, the buoy moved in the southeast direction of the anchor point, indicating that there was a strong shore current during this period. After 20:00 on that day, the east wind began to blow, and the buoy began to gradually shift to the west. Due to the combined effect of sea wind and shore currents, the buoy did not show a significant east–west shift until around 23:22 on 10 February, and the buoy began to shift to the southeast again.

### 3.2. GNSS Attitude Measurements

**Figure 7:**
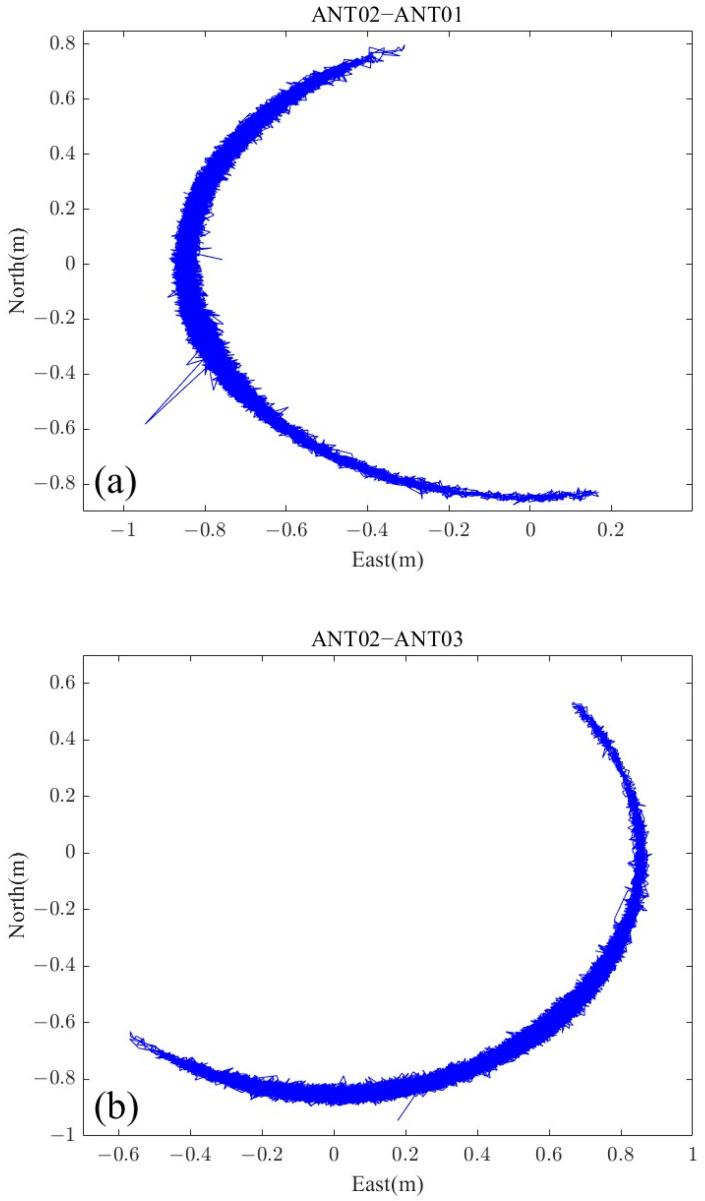

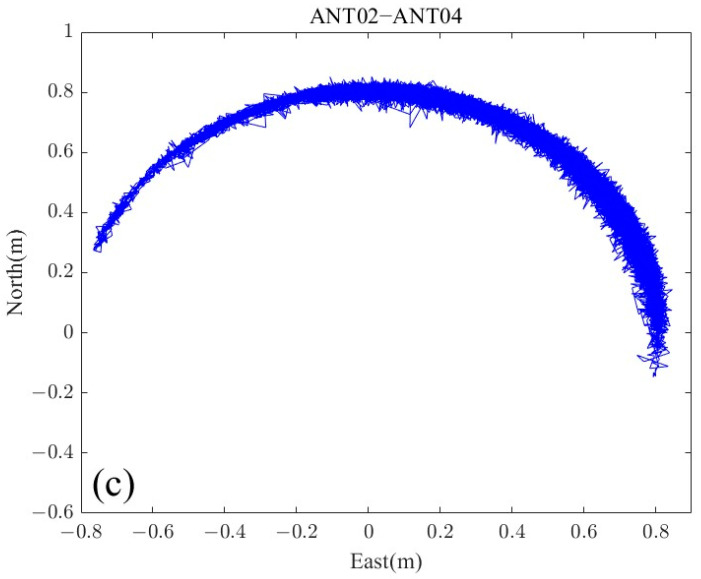
Dynamic baseline components in the eastward and northward directions: (**a**) baseline ANT02–ANT01, (**b**) baseline ANT02–ANT03, (**c**) baseline ANT02–ANT04.

The center antenna (ANT2) of the GNSS buoy was used to perform dynamic baseline calculations with the three other antennas to obtain the baseline vectors in the navigational coordinate system, and the data were obtained at a frequency of 1 Hz. The statistical results of dynamic baseline solution are shown in Table 3. The components of the three dynamic baselines of the GNSS buoy on 9 February in the eastward and northward directions are given in Figure 7, and there was a clear prominence in Figure 7a,b, which was due to unsuccessfully resolving the ambiguity of the baseline solutions.

**Figure 8:**
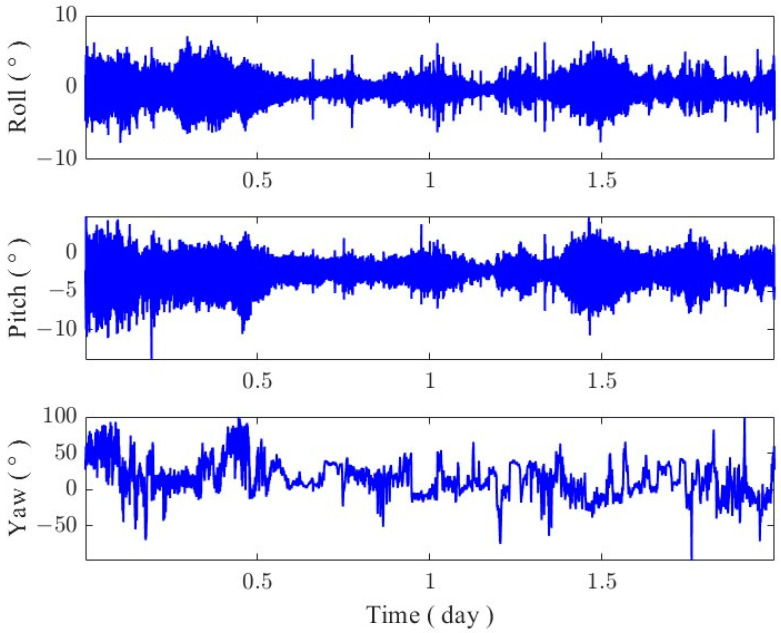
Three-axis attitude angle of GNSS buoys.

The attitude of the GNSS buoy was expressed as the attitude angles (roll, pitch, and yaw), which ranged from −90° to 90° for roll and pitch, and from −180° to 180° for the yaw angle. The three-axis attitude angles of the buoy solved using the multi-antenna GNSS technique are given in Figure 8, from which it can be seen that the magnitude of changes in the attitude angle of the buoy in the traverse and pitch directions were kept in the range of −10° to 10°, which indicates that the buoy was better stabilized and was better controlled in the traverse and pitch directions. In addition, it can be seen from the figure that the variation in the pitch angle had an overall deviation, which was due to the small deviation in the counterweight in the main bucket of the buoy, which had little influence on GNSS attitude calculation. In the follow-up experiment, the proportion of the counterweight should be further adjusted. At the same time, the bottom of the buoy was connected to the anchor with two mooring cables, so that the buoy will not rotate to a large extent in the horizontal direction; as can be seen in Figure 8, the change in the buoy’s yaw direction was mainly concentrated in the interval from −100° to 100°, and the buoy’s rotational angle did not completed a whole cycle.

The accuracy of the GNSS attitude solution depends on the accuracy of the dynamic baseline solution. It can be seen from the experimental baseline calculation results that in some epoch measurements, the errors reached the submeter scale, and the attitude calculation of the experimental buoy produced 5–8° errors. These errors occur because the GNSS ambiguity was not fixed correctly. To avoid such errors, more advanced fuzzy positioning techniques are needed, such as the use of additional observational data and improved algorithmic models.

### 3.3. Comparison of GNSS and Altimetry Radar Data

**Figure 9:**
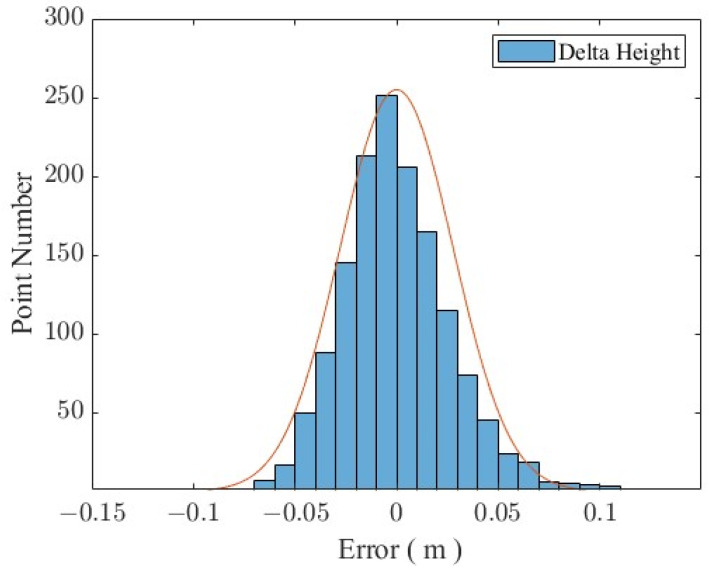
GNSS buoy and radar error statistics.

In this study, the TRG806X model altimetry radar produced by Dandong Top Electronics Instrument (Group) Co., Ltd. (Dandong, China), was used; the radar antenna is horn-shaped, and its measurement accuracy is ±3 mm, effective detection distance is 30 m, and data output strategy is the average value of 1 min. The small altimetry radar was fixed on an aluminum square tube. In order to reduce the influence of the after-effects of the waves hitting the pier on the radar altimetry, we extended the aluminum square tube fixing the altimetry radar to the pier by about 2 m, and the altimetry radar was about 6 m away from the sea level at the lowest sea level point. The aluminum square pipe was fixed onto the shore mounting bracket. A laser level was used to ensure the levelness of the radar altimeter’s mounting surface. Finally, several marble strips were placed on the bracket to provide stability. The statistical results of the sea surface height solved using the GNSS buoy data and the radar height measurement are given in Figure 9. The 95% confidence interval was approximately ±0.027 m, with a mean difference of 0.019 m, a standard deviation difference of 0.025 m, and an RMSE difference of 0.0254 m, which shows a good consistency and reliability.

**Figure 10:**
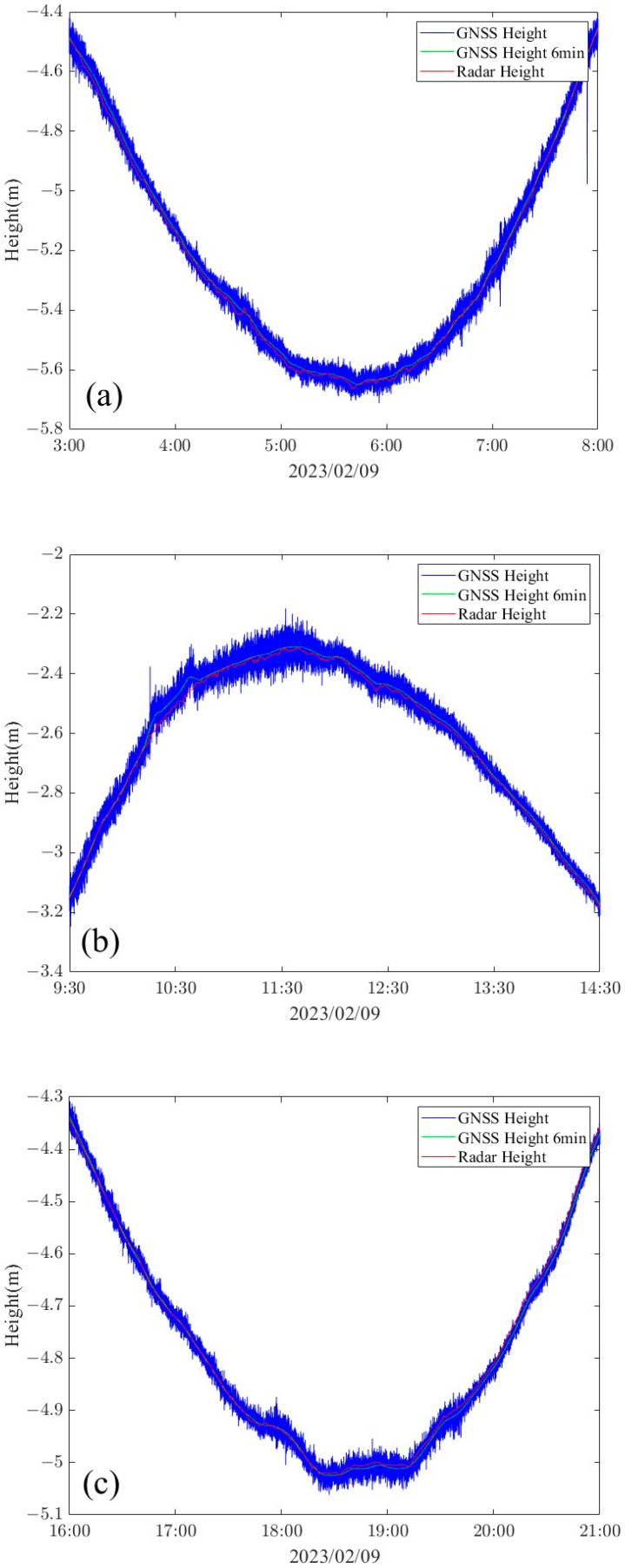

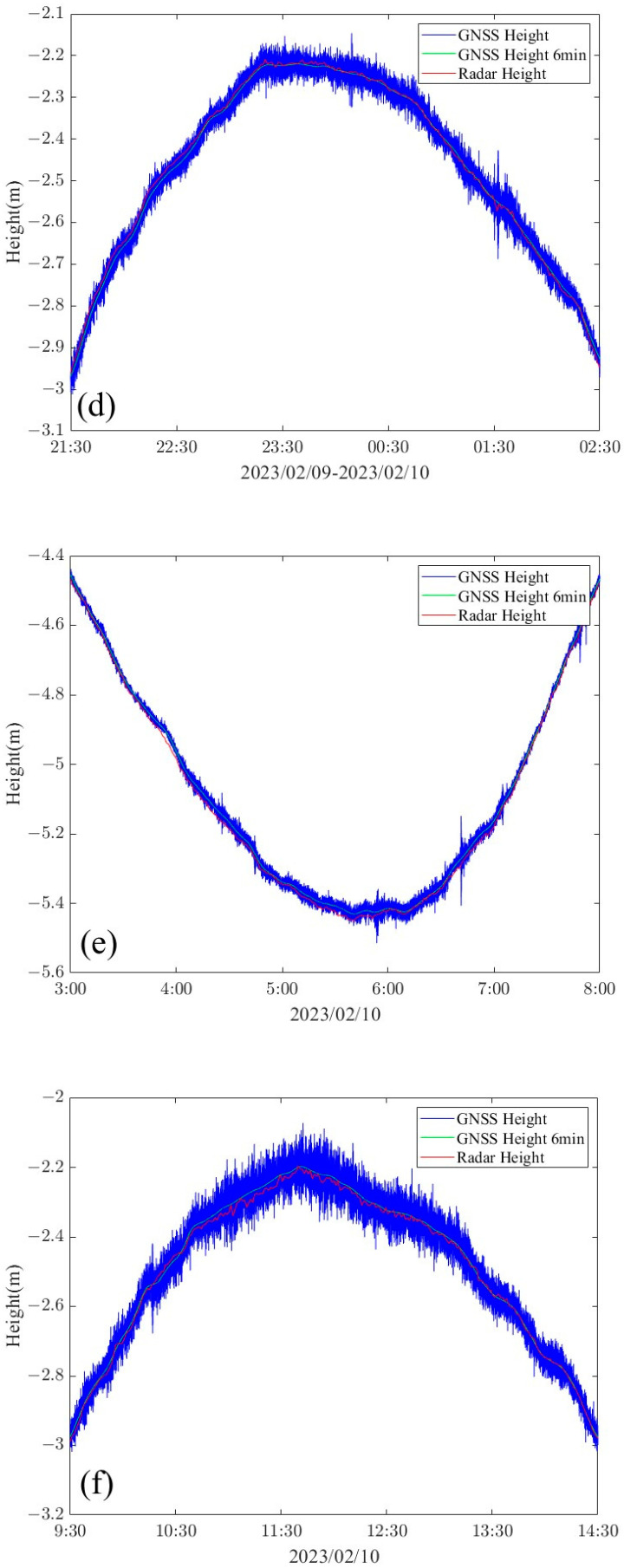

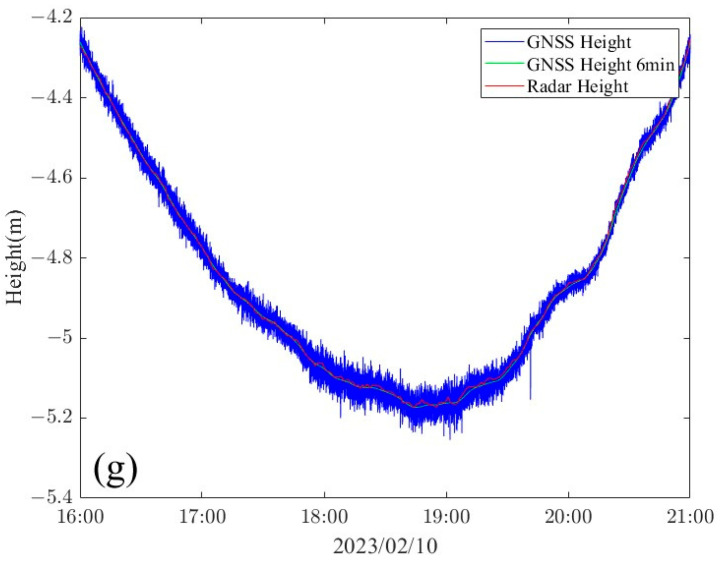
Sea surface height on (**a**) 9 February 2023 3:00–9 February 2023 8:00, (**b**) 9 February 2023 9:30–9 February 2023 14:30, (**c**) 9 February 2023 16:00–9 February 2023 21:00, (**d**) 9 February 2023 21:30–10 February 2023 2:30, (**e**) 10 February 2023 3:00–10 February 2023 8:00, (**f**) 10 February 2023 9:30–10 February 2023 14:30, (**g**) 10 February 2023 16:00–10 February 2023 21:00.

**Figure 11:**
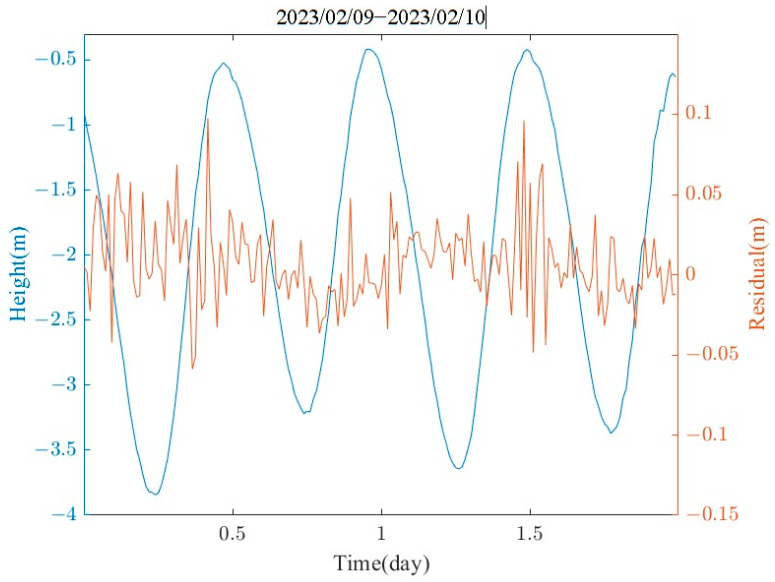
Comparison of 1 min interval resampled GNSS tidal observations with altimetry radar data as a baseline.

Seven complete high and low tides were recorded, and the data were statistically analyzed for the three-hour periods preceding and following each tide event (Figure 10). It can be clearly observed that the GNSS tide measurements generally corresponded with the radar altimeter observations in terms of overall trends. However, the GNSS elevation observations were subject to fluctuations due to the influence of ocean swells, sea breezes, and measurement noise. In particular, the measurement precision of the GNSS was significantly impacted during moments when the ambiguity of the GNSS was not resolved. A 900-s sliding window was used to denoise the GNSS buoy tide data, and the average value for each minute was calculated according to the sampling rate of the altimeter radar (1 min). The measured difference between the two methods is shown in Figure 11. The average difference between the GNSS and the altimeter radar was 0.0086 m. The standard deviation difference was 0.0111 m, and the RMSE (root-mean-square error) difference was 0.0115 m.

### 3.4. GNSS Attitude Correction Effect

**Figure 12:**
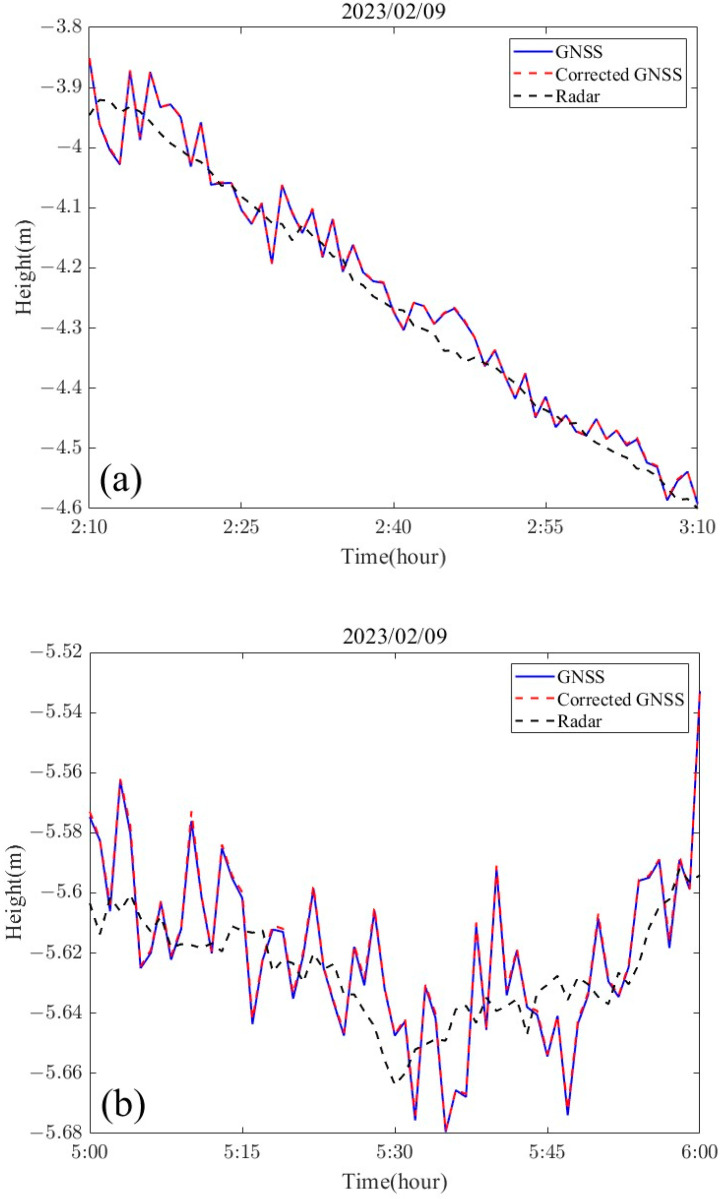

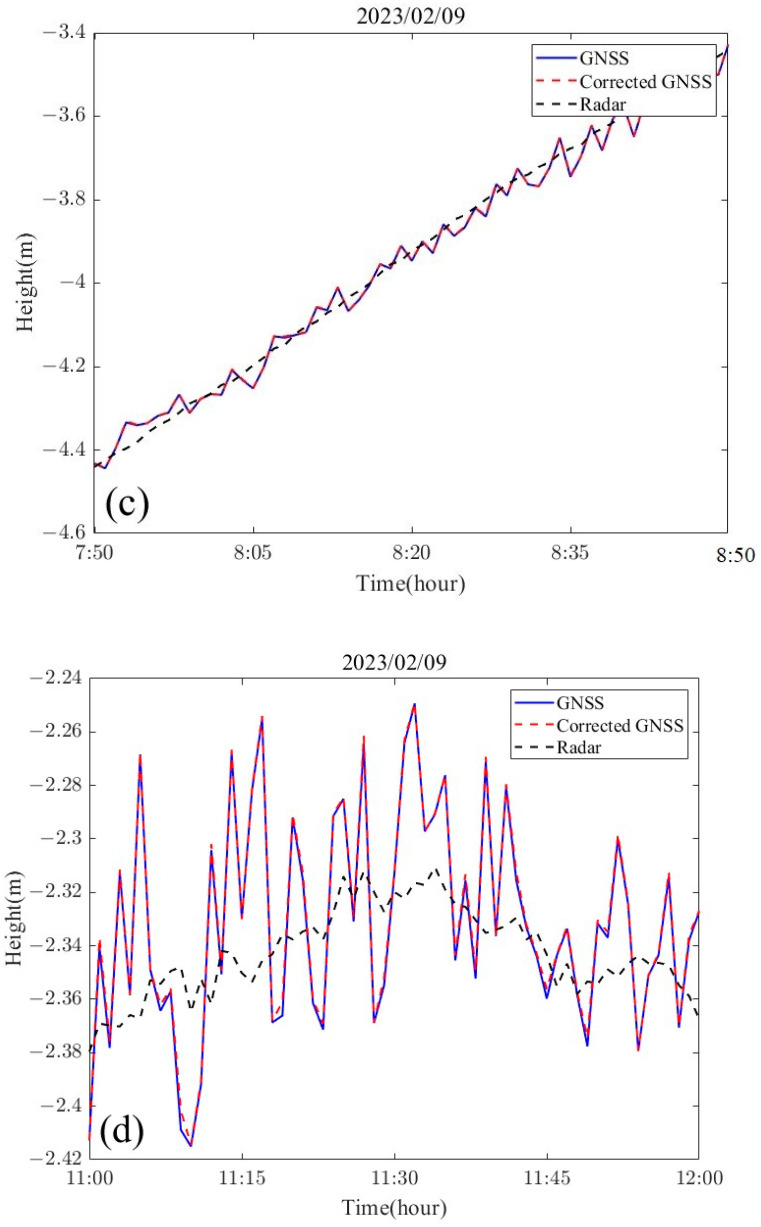
Effect of GNSS attitude correction, (**a**) low tide stage: 2:10–3:10, 9 February 2023; (**b**) lowest tide stage: 5:00–6:00, 9 February 2023; (**c**) high tide stage: 7:50–8:50, 9 February 2023; and (**d**) full tide stage: 11:00–12:00, 9 February 2023.

**Figure 13:**
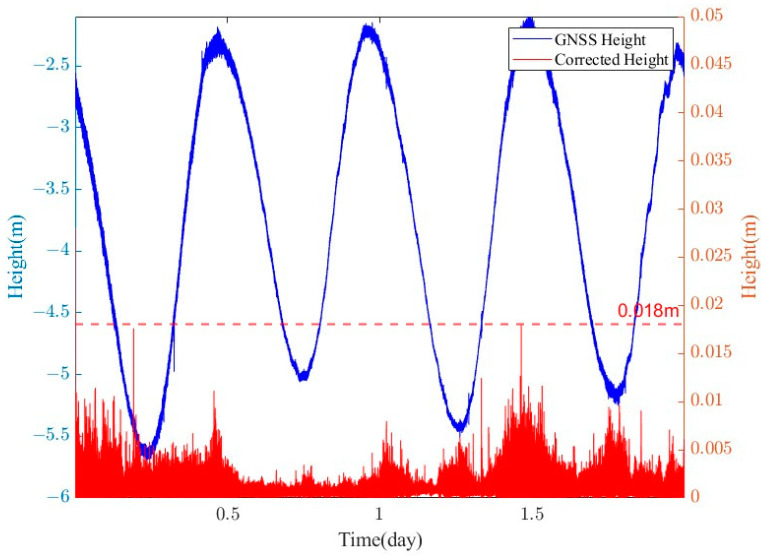
GNSS elevation time series with attitude modifications.

The main antenna of the GNSS buoy was located at the centerline of the buoy, and only the component of the lever arm value of the main antenna in the vertical direction needed to be calibrated (0.61 m). Figure 12 shows the attitude corrections for four different tidal stages. The buoy’s attitude tilt correction was minimal and essentially consistent with the original data, and from the statistics of the whole observation period (Figure 13), the tidal level measurement error caused by the attitude perturbation was within 1 cm, while the highest achievable correction given in the figure was up to 1.8 cm. The reason for this situation is due to the poor quality of the dynamic baseline solution (the ambiguity was not fixed), which caused errors in the estimation of the attitude angle. The accuracy statistics using a single GNSS antenna and after correction using the multi-antenna GNSS are given in Table 4. The mean difference, standard deviation, and RMSE of the tidal time series after attitude correction were 0.0188 m, 0.0250 m, and 0.0252 m, respectively, which were reduced by 0.1 mm, 0.1 mm, and 0.2 mm, respectively, compared to the uncorrected GNSS tidal time series. The mean difference, standard deviation, and RMSE of the two methods after 900 s sliding window processing were reduced by 0.4 mm, 0.5 mm and 0.3 mm, respectively, after correction compared to before the correction.

## 4. Conclusions

In order to investigate the application of multi-antenna GNSS technology in GNSS tide observations, a set of low-cost offshore buoys carrying four GNSS antennas was designed and deployed for testing in the offshore area of Jimiya Wharf in Qingdao City, China, from 00:00 on 9 February 2023, to 00:00 on 11 February 2023 (UTC), and the raw observation data at 10 Hz from the four GNSS antennas were collected.

(1) The PPK positional calculation was performed on the center antenna of the buoy (Figure 11), and seven complete high-water and first-water levels were obtained. The comparison with a small surface side-cover radar with simultaneous observations yielded a tidal measurement accuracy of about 1 cm (RMSE of 1.12 cm) for the GNSS buoy, which is similar to that obtained from previous deployments of similarly structured GNSS buoys [34], and the remaining measurement error of 1 cm was related to the performance of the GNSS technology and receiver hardware.
(2) The data from the four antennas mounted on the GNSS buoy were processed to obtain the attitude time series of the buoy (Figure 8), and it was found that the magnitude of the change in the attitude angle in the roll and pitch directions in the sea of the GNSS buoy maintained a high degree of consistency, which also verified the previous conclusions of the attitude measurements using the GNSS/INS method [21]. From the results of the attitude solution, the normal high attitude correction for the instantaneous sea surface height on our designed buoy was within 1 cm (Figure 12), while a small amount of correction exceeding 1 cm existed, but these higher corrections were not based on the attitude angle corrected from the normal solution, but were due to the unfixed ambiguity in the dynamic baseline solution process leading to the error in the attitude angle solution [35,36].
(3) After correcting the tidal data of the GNSS buoy using the attitude angle measured by the multi-antenna GNSS, the tidal measurement accuracy was only improved by 0.3 mm compared to the pre-correction values, and similar problems have been encountered by scholars in previous studies [37], which occurred for two reasons: first, because the wobble angle of the buoy itself is not large (−10°~10°), and the second is that the distance of the main antenna of the GNSS buoy from the center of the buoy was very short (0.61 m), which, under the combined influence of these two factors, leads to a small amount of attitude correction for the GNSS buoy, which was not prominent in the tidal extraction process.

By trying to carry multiple GNSS antennas on the GNSS buoy, the attitude measurements of the buoy and the observation of tides were successfully realized. In this study, considering the influence of the attitude of the buoy on the measurement of the sea surface height, the effect of the attitude correction was not outstanding due to the low height of the main antenna at the center of the buoy, although it was tested and verified on the self-designed multi-antenna buoy. In order to further validate the effect of attitude correction on tide level measurement accuracy, future research will include plans for experiments on large offshore buoys to more comprehensively assess the effect of attitude correction on the improvement of sea surface height measurement accuracy of large ocean buoys. In addition, the tidal observation factors affecting the GNSS buoys are not only attitude changes, but also the effects of the buoy rising and sinking in the vertical direction, which needs to be taken into account in subsequent studies.

## Figures and Tables

**Table 1:** Multi-antenna GNSS buoy design dimensions.

Parameter	Parameter Indicators
weight	113.5 kg
Height	946 mm
Main radius	250 mm
Max buoyancy	1747.096 N
Max load	55 kg
Depth of entry at full load	400 mm
Volume of the main body of the buoy	560 mm × 250 mm × 250 mm

**Table 2:** Calibration results of relative elevation.

Calibration Position	Calibrated Elevation
Total station control point elevation	0 m
Total station instrument elevation	1.365 m
GNSS base station height (antenna phase center)	1.5921 m
Sea surface altimetry radar antenna height	0.151 m
Total draft of the buoy	0.799 m

**Table 3:** Precision statistics of dynamic baseline solutions.

Baseline	Baseline Length	Ambiguity Fixed Rate	Standard Deviation of Baseline Length	Max Error
ANT02–ANT01	0.9668 m	96.5%	0.0042 m	0.3305 m
ANT02–ANT04	0.9698 m	97.3%	0.0031 m	0.2705 m
ANT03–ANT04	0.9682 m	97.9%	0.0028 m	0.2518 m

**Table 4:** Comparison of accuracy of multi-antenna GNSS buoy tide level measurements.

Method	MAD	SD	RMSE	Max Error
GNSS	0.0189 m	0.0251 m	0.0254 m	0.1546 m
Corrected GNSS	0.0188 m	0.0250 m	0.0252 m	0.1518 m
GNSS (900 s)	0.0086 m	0.0111 m	0.0115 m	0.0479 m
Corrected GNSS (900 s)	0.0082 m	0.0105 m	0.0112 m	0.0477 m

## Data Availability

The data presented in this study are available on request from the corresponding author.
